# Philadelphia Translocation in MDS: A Case Report and a Brief Review of the Literature Looking at Its Prevalence, Disease Progression, and Treatment Options

**DOI:** 10.1155/2018/5865321

**Published:** 2018-11-22

**Authors:** Lakshmi Ramya Chelapareddy, Sandeep Sen

**Affiliations:** SSM Health St. Mary's Hospital, St. Louis, Missouri 63117, USA

## Abstract

Myelodysplastic syndrome (MDS) is a group of clonal disorders characterized by ineffective and dysplastic hematopoiesis in the bone marrow with variable risk of progression to leukemia. MDS is characterized by specific karyotypic and molecular abnormalities. The t(9 : 22) Philadelphia translocation is not a common abnormality found in MDS, and it is not included in the prognostic indices for germline mutations. There are no definitive treatment guidelines for these patients either. Here, we reviewed previously reported cases of MDS with the Philadelphia translocation with a goal to determine their prognosis and treatment options, specifically the tyrosine kinase inhibitors (TKIs).

## 1. Introduction

MDS is a group of acquired disorders characterized by ineffective and dysplastic hematopoiesis in the bone marrow with variable risk of progression to leukemia. MDS can either be de novo or develop after mutagenic therapy or environmental exposure to toxins, radiation, and chemotherapeutic agents. Symptoms are usually nonspecific, and some patients remain asymptomatic for a long time before diagnosis. Symptoms are based on the cell line involved and can present as fatigue, weakness, dizziness, confusion in case of anemia or infections in neutropenia, or bleeding due to thrombocytopenia or dysfunctional platelets. Philadelphia chromosome is a translocation involving the chromosomes 9 (Abelson protooncogene/ ABL) and 22 (breakpoint cluster region/ BCR) with a resultant fusion oncogene which encodes the BCR-ABL protein with enhanced ABL1 activity. It is a disease-defining entity in chronic myeloid leukemia (CML) and is seen in acute lymphoblastic leukemia (ALL) as well but is rarely reported in patients with MDS, and its presence on prognosis or management is not defined. We report a case of MDS with the Philadelphia translocation here and reviewed other similar cases.

## 2. Case Report

A 83-year-old Caucasian male with a past medical history of TIA and nephrolithiasis initially presented for hematologic evaluation of persistent leukocytosis with immature cells on peripheral smear found on routine office visit. On initial evaluation, peripheral smear showed a leukocytosis of 17,500, with neutrophil predominance of 72%, lymphocytes 10%, monocytes 13%, eosinophils 1%, myelocytes 1%, and blasts 3 %. Absolute neutrophil count (ANC) was 2600 cells/mcL, along with mild anemia, hemoglobin of 11.6 mg/dl, and thrombocytopenia 90,000. His metabolic profile was within normal limits. The patient was asymptomatic except for easy fatigability for few months. No B symptoms were present on initial evaluation.

Initial evaluation with ANA, RA factor, ESR, CRP, iron, ferritin, thyroid function tests, vitamin B12, folate, copper serum, and urine immunofixation tests was normal.

Bone marrow biopsy was done due to suspicion for CML vs. MDS which showed hypercellular marrow (80% cellularity) with granulocytic hyperplasia and trilineage dyspoiesis with <5% blasts. Fluorescence in situ hybridization (FISH) performed on bone marrow was negative for BCR-ABL fusion gene—p190 and p230 isoforms—and was negative for MDS/ AML probes—PDGFRA/ PDGFRB/ FIP1L1/ CHIC2 negative. Cytogenetics was normal with analysis showing 46,XY with no evidence for any clonal structural or numerical abnormality.

Blood CML PCR quantitative panel was negative for b2a2 and b3a2 (p210) and E1a2 (p190) transcripts along with JAK2V617F mutation.

The patient was diagnosed as low-grade (IPSS-R score 2.5, low risk) myelodysplastic syndrome (MDS) and was monitored as he was mostly asymptomatic and had been doing well. 10 months later, he developed progressive symptomatic anemia, with hemoglobin drop to 8.8 from a baseline of 11.6. Meanwhile, his leukocytosis resolved and thrombocytopenia seemed to improve to >100,000. The patient was started on weekly Epoetin alfa with improvement in his anemia. Leukocytosis resolved, but 8 months later, the patient developed significant thrombocytopenia with platelet count drop from 121,000 to 64,000 in 1 week. Workup for thrombocytopenia was initiated with slightly elevated prothrombin time 11.8 sec, INR 1.2, and normal PTT. Von Willebrand Factor antigen and Factor III activity were normal. A repeat bone marrow biopsy showed hypercellular marrow for age (approx 80% cellularity), with trilineage hematopoiesis with significant dysmegakaryopoiesis and mild dyserythropoiesis. No evidence of T or B cell lymphoma or acute leukemia was found.

Flow cytometry on bone marrow specimen revealed neutrophil predominant specimen with 74% neutrophils, monocytes 3%, lymphocytes 10% with normal subtypes, and 2.9% blasts. Cytogenetics on bone marrow with analysis of 100 interphase cells revealed new BCR-ABL1 fusion gene t(9; 22)(q34; q11.2) with translocation between 22q11.2 and 9q34 in 79% of cells. Peripheral blood RT PCR for BCR-ABL was positive for BCR-ABL p210 transcript (b2a2 at 11.042%). Pt was diagnosed as Ph+ve MDS/MPN, intermediate risk and was started on tyrosine kinase inhibitor imatinib which was discontinued 7 months later due to lack of significant decrease in Ph+ve clone on repeat marrow biopsy. He was instead started on dasatinib which was discontinued 1 week later due to development of right sided heart failure. He did not have a T315I mutation and was not a candidate for ponatinib. He is currently doing well, 30 months after diagnosis, not requiring transfusion support and is currently waiting for nilotinib approval. His last BCR-ABL level was 9.9% in peripheral blood at last check 1 month ago.

## 3. Discussion

MDS is a clonal process thought to arise from a single transformed hematopoietic progenitor cell [1, 2]. The incidence of MDS is variable, but it is estimated to be about 10,000 cases per year, usually diagnosed after the age of 50 [3, 4]. Common cytogenetic abnormalities associated with MDS include +8, loss or del of chromosomes 5 or 7, del 20q [5]. The BCR-ABL fusion gene is a disease-defining clonal abnormality, usually seen in CML (in >95%) and in some cases of ALL (17–25%) [6]. MDS is rarely associated with BCR-ABL mutation and is usually reported at the time of progression to acute leukemia [7]

A consensus prevalence of the BCR-ABL fusion gene in patients with MDS is currently unavailable. In the study by Keung et Al, the prevalence seems to be about 2% of cases, but this did not differentiate between de novo and treatment-related MDS [8]. A further literature search revealed a total of 22 cases of MDS with the Philadelphia chromosome. Details of patient characteristics are shown in Table 1, [8–25]. Patients with Philadelphia chromosome like mutations and patients with acquisition of the BCR-ABL gene at the time of evolution to acute leukemia have not been included in the table [26–28].

The Philadelphia chromosome is formed by the translocation of t(9 : 22) with a resultant fusion gene which encodes the oncoprotein BCR-ABL1, with enhanced ABL tyrosine kinase activity [29, 30] leading to increased proliferation of myeloid cells which could lead to transformation of these patients with MDS into acute leukemia. It is estimated that about a third of MDS cases diagnosed will transform into AML, and the BCR/ABL1 translocation is estimated to be present in approximately 1% of patients with AML [7]. Considering the appearance of the Philadelphia chromosome in patients after or at the time of evolutions to leukemia [20, 26–28], it might indicate an overall poorer prognosis.

In addition to known prognostic factors, like cytopenia, bone marrow blast percentage, and karyotype [31], we looked to see if presence of the Philadelphia translocation would lead to increased risk of transformation.  Table 2 demonstrates the number of events of progression versus no progression. Of the patients with available data, *n*=15, more than 50% (*n*=8) patients transformed to leukemia (including AML, CMML, CML, and myeloid sarcoma) which is much higher than the predicted risk of transformation. 8 of the patients received TKIs, with variable response, but they seem to perform worse with conventional chemotherapy or just supportive care with death due to sepsis being the common reason for mortality. One patient had rising blast count with conventional chemotherapy but responded to imatinib [15]. Overall, patients seem to have a poorer prognosis with increased transformation to leukemia compared to patients without BCRABL mutation and do not respond to conventional chemotherapy or supportive care, although they seem to have a semblance of response with the tyrosine kinase inhibitors.

It is difficult to determine the exact incidence or prognosis of patients with MDS in whom this mutation is found at this point due to the rarity of its presence, but it would be imperative to suggest that these patients have increased risk of proliferation and probably transformation into leukemias. Routine testing for the Philadelphia translocation is not a part of the NCCN recommendations [32], and it is not part of the MDS panel probe testing for molecular genetics and is not listed in the common gene mutations in NCCN, but this translocation can be identified with conventional cytogenetics and when present, consider treating with tyrosine kinase inhibitors. TKI resistance can occur after treatment with TKIs and checking for T315I mutation in these patients is recommended to switch therapy.

Furthermore, a national database to study the incidence of these mutations and study overall survival and prognosis would be helpful in the future.

## Figures and Tables

**Table 1:** Patient characteristics, cytogenetics, time to progression, treatment outcomes, and bone marrow blast percentage

Blast count (patients)	<10% (*n*=11)	10–20% (*n*=7)	>20% (*n*=2)	Unavailable (*n*=3)
No progression	5	2	0	1
Leukemic transformation	5	3	2	1
No data	0	1	0	1
RAEBt	1	1	0	0

**Table 2:** Blast count and number of events.

Age	Gender	Classification	Cytogenetics	Bm blast%	Plt/mcL	Hgb g/dL	Time to progression	Treatment	Outcome	Reference
74	M	RCMD	44, XY, t(9 : 22) [4], 46,XY[16]- 46%	0	341	8.3	No progression	Dasatinib	Hospice after 7m following sepsis	[9]
55	F	RAEB I	45XX,−4,t(9; 22)(q34; q11.2)	6	10	10.3	No progression	Supportive care	Died in 1 month sepsis	[10]
31	M	RAEB II	44–45XY,der(4),−5,der(7),−7,−8,t(9; 22)(q34; q11.2),−15,+22	14	54	4.7	No progression	Supportive	Died in 1 week - IC bleed?	[10]
65	M	RAEB 2	44xy del 5,-7,-14,-16 + mar	18	n/a	n/a	No progression	None	Died in 1 m from lung cancer	[11]
67	M	RAEB1-> RAEB 2	45,XY,+3,−5,−7,−20,+mar,+mar/45,XY,	4	52	10.4	No progression	Imatinib for 2 weeks f/b supportive care	Died 1 yr from fungal pneumonia	[12]
			+3,−4,−8,−9,−11,−18,−20,−21,−21,+mar,+mar							
61	M	MDS unclassifiable	46,XY,der(5; 12)(q10; q10)+mar[3]/46,XY[12] t(9; 22)by FISH in der(5; 12)	n/a	n/a	n/a	n/a	Imatinib, hydroxyurea, cytarabine	Died in 5m from resp failure	[13]
71	M	RAEB	46XX,der(12)t(12; 17)(p11.2; q11.2)[7]/46, XX[9]	10	54	9	To RAEBt in 5 m	Hydroxyurea	Died in 4 m from pneumonia	[14]
66	F	RAEB	47,XX,+8, t(9; 22; 16)(q34; q11.2,q23)[4]	2	275	7.5	Progressed to granulocytic sarcoma of skin in 9 m	Supportive care	Died 1 m later	[14]
59	M	RAEB	46,XY,t(9; 22)(q34; q11)	4	78	9.2	4 m to AML	Imatinib	Alive after HCT	[14]
67	F	RAEB 2	46,XX[1]/46,XX,t(9; 22)(q34; q11)[29]	10	114	11.5	NA	Conventional chemotherapy with rising blast % responded to imatinib.	Alive at 14 m after imatinib	[15]
73	M	RAEB	46 XT t(9 : 22),	8	n/a	n/a	CML in 2.5 yr f/b blast crisis in 3 m	Imatinib	Died with refractory CML and pneumonia	[16]
78	M	RAEB t	46,XY der (3), t(1 : 3)(p22:p14), t(9; 22)	22.2	29	9.8	n/a	Ara-C+daunorubicin->AraC+IDarubicin	Died from pneumonia 5 m after Dx	[17]
69	M	RAEBt	+Y, del5,q13q34,+8,+13,+14, p11+22, t922q34	14	46	8.1	2 m to AML	Vindesine f/b cytosine arabinoside, mitoxantrone, tenoposide	Died from pneumonia 5 m after Dx	[18]
64	M	RAEB	+8, t(9 : 22)(q34)- 46%	4	98	7.8	9 M to AML	Hydroxyurea f/b cyt ara-c, teniposide	Died from sepsis 9 m after Dx	[18]
30	M	MDS	46,XY[1]/46,XY,t(9; 22)(q34; q11)[45]	0.5	30	12.5	No progression	n/a	Died 2 years after due to intracranial bleeding	[19]
78	F	RAEB 2	46 XX, t(9; 22)(q 34; q 11)	11.7	21	8	5 m to AML	Transfusion, ubenimex	Died from pneumonia	[20]
73	M	MDS	46,XY[54]/46,XY,t(4; 6)(p15; p12),t(9; 22) (q34; q11)[6]	1	120	15.4	7 m to CMML	Supportive care	Died from bleeding 10 m after dx	[21]
85	M	RAEB	46,XY,t(9; 22)(q34; q11)	25	120	9.8	17 m to AML	Daunorubicine, cytarabine, 6-mercaptopurine. Prednisone f/b 6-mercaptopurine, hydroxyurea.	Died 21 m later	[22]
62	M	MDS	46,XY[?]/t(?; 11; 22)(?; q11; q11),t(9; 22)(q34; q11), −5,−7,+8,−12	8.2	3	11.9	3 m to RAEBt	Prednisolone and vit D3	Died 3 m later from pneumonia, bleeding	[23]
70	FM	RARS	46,XX[3]/46,XX,t(9q; 22q)[12]	n/a	316	9.5	No progression	Pyridoxine, nandrelone, folinic acid	Alive at 45 months	[24]
49	F	MDS	46 XX (3) t(9 : 22) 34 : 11	n/a	425	8.2	32M TO AML	Ara-C, 6-thioguainine, daunomycin	Died from pneumonia 1 m after AML	[25]
78	F	MDS-RARS	46,XX--> 46,XX,t(9; 22)(q34; q11)	14	152	10.2	26 m to CML blast crisis	Imatinib and nilotinib	Hematologic response on TKI	[26]
83	M	MDS-RCMD	46 XY--> 46,XY,t(9; 22)(q34; q11.2)[10]/46,XY[5]	<1	90	11.6	No progression	Imatinib-->dasatinib->nilotinib	Alive after 30 months	Case

M = male, F = female, RCMD = refractory cytopenia with multilineage dysplasia, RAEB = refractory anemia with excess blasts, RARS: refractory anemia with ringed sideroblasts, CML= chronic myeloid leukemia, CMML= chronic myelomonocytic leukemia, BM blast = bone marrow blast %, and Plt = Platelet.
